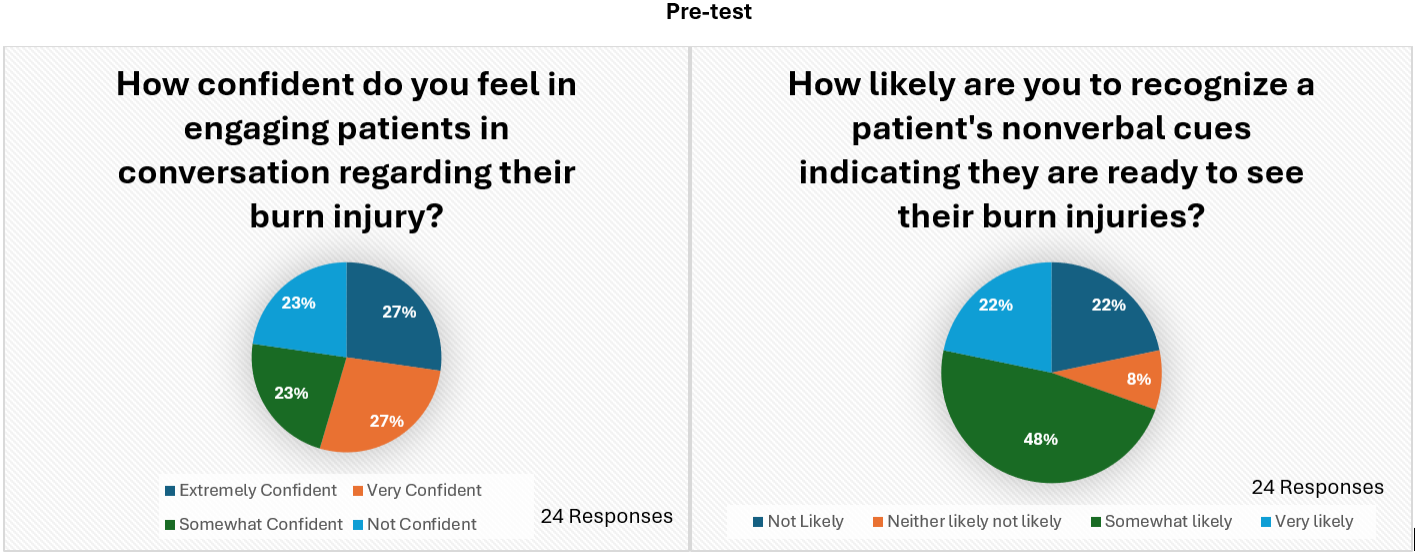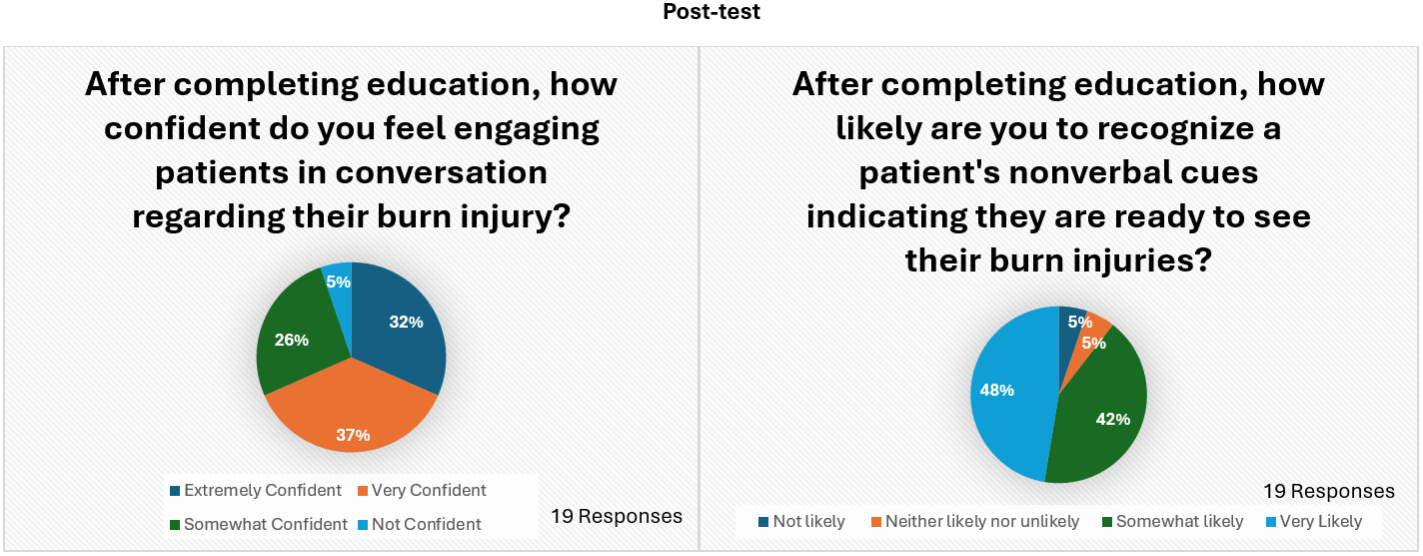# 7 The First Look: Nursing Empowerment with Helping Self-Image of Burn Survivors

**DOI:** 10.1093/jbcr/iraf019.007

**Published:** 2025-04-01

**Authors:** Christine Steen, Erika Gonzalez, Brooke Dean, Tessa Welch, Lisa Smith

**Affiliations:** Johns Hopkins Bayview Medical Center; Johns Hopkins Adult Burn Center; Johns Hopkins Bayview Medical Center; Johns Hopkins Bayview Medical Center; Johns Hopkins Bayview Medical Center

## Abstract

**Introduction:**

Patients seeing their burn injuries for the first time can be a traumatic experience evoking responses such as anxiety, fear, or grief. Burn nurses play a key role in preparing survivors for their transition to life outside of the hospital through crucial conversations. Nurses revealed that they felt competent in providing wound care yet unprepared to have difficult conversations with patients during the first look at their burn injuries. This project’s purpose is to develop training for nurses that would strengthen knowledge and communication skills with patients.

**Methods:**

A pre/post-test was administered to nurses to self-evaluate their competencies supporting patients, engaging in difficult conversations, and recognizing non-verbal cues during their first look. A burn survivor was invited to share their journey in burn recovery and offer feedback on their experiences to enhance the staff’s understanding of patient perspectives. A presentation on social skills empowerment to help patients craft their response when approached about their injuries was provided to staff. Burn Survivor Peer Supporters provided an overview of resources for survivors, including podcasts, events, and opportunities to connect with peers.

**Results:**

The pre-test revealed that 23% of nurses were not confident in engaging patients in difficult conversations about their burn injuries, while 27% felt very confident. Additionally, 22% of nurses believed they were unlikely to recognize nonverbal cues, with an equal percentage believed they were likely to do so. Following training, only 5% of nurses still lacked confidence in handling difficult conversations, while the percentage of those feeling very confident rose to 37%. Results also showed that 5% of nurses remained unlikely to identify nonverbal cues, while 48% felt very confident in their ability.

**Conclusions:**

Disfigurement can lead to profound grief and distress requiring comprehensive care and support. Formal training sessions designed to help nurses recognize non-verbal cues and facilitate difficult conversations can increase nursing confidence in these skills.

**Applicability of Research to Practice:**

Beyond performing wound care and other tasks, nurses must be equipped with the knowledge and skills to effectively support the overall well-being of their patients. This training empowers nurses to provide effective emotional and psychological support and better patient outcomes by fostering a more supportive environment for healing.

**Funding for the Study:**

N/A